# Robust rateless space time block coding for mmWave massive MIMO system

**DOI:** 10.1016/j.heliyon.2024.e40114

**Published:** 2024-11-02

**Authors:** Zelalem A. Kelem, Habib M. Hussein

**Affiliations:** Artificial Intelligence and Robotics Center of Excellence, Addis Ababa Science and Technology University, Department of Electrical and Computer Engineering, Ethiopia

**Keywords:** Wireless communication, Massive MIMO, Rateless space-time block coding (RSTBC), Orthogonal space time block codes (OSTBC), Rateless orthogonal space time block codes (ROSTBC)

## Abstract

In the vast growing wireless communication system creating robust encoding mechanisms using space-time block codes (STBC) for mmWave massive MIMO to overcome uncertainty and ensure reliability is a critical concept to be covered. This article reviews the core concepts behind MIMO communication, Massive MIMO communication, space-time block codes, and rateless codes in the context of wireless communication systems. Building on the foundational concepts of information theory and mmWave massive MIMO, we developed space-time block codes that maintain orthogonality for real-valued symbols. Following a thorough analysis of these codes, we successfully extended them into rateless orthogonal space-time block codes (ROSTBC) for massive MIMO. This extension enables the codes to adapt their rates dynamically based on the unknown channel conditions at the transmitter. The research work was concluded by comparing static G4 encoded Tarokah work and other Orthogonal Space Time Block Codes (OSTBCs) with Rateless Space Time Block Codes (ROSTBCs). The results show that as the number of blocks used to encode the message in Rateless Space Time Block Code (ROSTBC) increases, this coding scheme can outperform static Rateless Space Time Block Codes (OSTBC) in very low SNR values by a minimum of 8.5 %. This work can enhance wireless communication by creating reliable communication over inherently unreliable systems.

## 1 Introduction

The evolution from MIMO technology then to Massive MIMO represents a significant advancement in wireless communication systems, driven by the need to enhance spectral efficiency, increase data rates, and improve overall network performance. Massive MIMO addresses this challenge by allowing the reuse of the same frequency spectrum for multiple users, increasing the overall network capacity and supporting higher data rates.

The world we are in requires a good quality of service with ensured user experience. However, the challenge of fitting this issue to the concept of wireless communication is always a headache for communication Engineers. Many methods have been proposed by researchers to mitigate the challenges of wireless communication in creating reliability. One of the proposed technologies was to use a combination of spatial and temporal processing known as space-time block code (STBC) [1].

Previous research has shown that space-time block codes (STBC) using lower modulation orders consistently yield lower bit-error rates compared to those using higher-order modulation techniques. Simple linear maximum likelihood (ML) detection and decoding have been found to be more efficient than joint detection and uncoded SISO, as demonstrated in research [2]. Building on earlier work conducted on AWGN channels, rateless codes for MISO Gaussian channels have been successfully designed using techniques such as dithering, layering, and repetition, and these were combined with STBC for MISO antenna configurations [3]. Additionally, tests across various channel conditions and message sizes revealed that spinal codes significantly outperform fixed-rate LDPC codes, rateless Raptor codes, and Strider's layered rateless coding strategy in terms of throughput [4].

Further studies have highlighted the effectiveness of rateless space-time codes, particularly in challenging scenarios. For instance, Ali A. Alqahtani demonstrated that these codes are efficient even when facing a data loss rate of 25 % or higher [5]. Similarly, Raptor rateless codes, encoded with a linear-time progressive edge growth algorithm and using joint iterative decoding methods, have been optimized for deep space communications to ensure good decoding performance [6]. Moreover, research indicates that in massive MIMO systems with numerous antennas, some antennas may fail, yet RSTBC can still maintain robust performance, even with up to a 25 % antenna loss rate [7].

Additionally, research made by Ref. [21] investigated UAV-enabled massive MIMO-NOMA relay systems with low-resolution ADCs/DACs, deriving closed-form expressions for the sum spectrum and energy efficiency (SE/EE) under imperfect CSI, SIC, and quantization noise. This gave emphasis on the impact of system parameters like power scaling and imperfect conditions on efficiency. A recent work of [22] on intelligent reflecting surfaces based offset index modulation for MIMO systems shows transmission reliability and spectrum efficiency can also be improved by leveraging innovative approaches such as intelligent reflecting surface (IRS) elements.

MIMO systems improve the capacity and resilience of wireless networks, making millimeter-wave communication an attractive technology for future wireless networks with its potential for high data rates and greater bandwidth. However, the inherent challenges of millimeter-wave propagation, including CSI accuracy, path loss, and susceptibility to atmospheric absorption, necessitate robust error mitigating and channel encoding mechanisms to ensure reliable communication.

Earlier studies reveal significant research gaps, particularly in obtaining accurate CSI in dynamic channel environments. As the number of antennas increases, the accuracy of CSI knowledge tends to decline, further complicating the system design. Additionally, the integration of space-time block codes with fountain codes for larger antenna configurations, while ensuring orthogonality during transmission, remains underexplored. Previous research on rateless STBC primarily focused on erasure channel modeling and mitigating antenna failures using this encoding scheme, but further exploration is needed to address these challenges comprehensively. The aim of this article is to leverage the capabilities of rateless coding and Orthogonal Space-Time Block Codes (OSTBC) to address the critical challenge of acquiring accurate channel state information (CSI) in mmWave Massive MIMO systems, thereby enhancing communication reliability under dynamic channel conditions.

## 2 Mathematical modeling

### 2.1 Modeling MIMO communication system

To effectively model a MIMO communication system, it is important to grasp the underlying mathematical framework. Imagine a MIMO system with $N_{T}$ Transmit antennas and $N_{R}$ Receive antennas. Given that the average transmit symbol, energy is Es, the output-input relation between the transmitted and received signals can be illustrated as follows [8]:

(1) $$Y\left[k\right]=\sqrt{\frac{Es}{N_{T}N_{o}\;}}\;H\;S\left[k\right]+n\left[k\right]$$

where S[k] is the transmitted symbol vector with dimension $N_{T}\;x\;1$ for K number of samples taken, H is $N_{T}$ x $N_{R}$ Matrix which represents the channel matrix. n[k] is the $N_{R}\;x\;1$ spatial-temporal zero mean, complex Gaussian white noise vector with variance $N_{o}$ and Y[k] is the received signal vector with dimension $N_{R}\;x\;1$.

If we rewrite eq (1) by removing the sampling (time index) K for clarity we will have [8],

(2) $$Y=\sqrt{\frac{Es}{N_{T}N_{o}\;}}\;H\;X+n$$

Since the transmitter is usually power-limited, we can put an average power constraint in the transmitted signal. $X_{k}$. So, the average power can be defined as the covariance matrix of **S**, which is given by,

(3) $$R_{ss}=E\;\left\{{XX}^{H}\right\}$$

Superscript $\prime^{H^{\prime }}$ Represents the Hermitian Operation which is also called conjugate transpose of the matrix and E[.] Is the expected value operation. Let's assume the channel matrix H is deterministic and known to the receiver, so that channel state information is known on the receiver side. This can be achieved by sending pilots, conducting experiments, or sending feedback. Then the capacity of the MIMO channel can be given as [9].

(4) $$C=\max_{T_{r}\left(R_{\text{ss}}\right)=N_{T}}W\;\log\det\left[I_{N_{R}}+\frac{E_{s}}{N_{T}\;N_{o}}\;HR_{\text{ss}}\;H^{H}\right]$$

### 2.2 Modeling STBC for MIMO communication

Block codes in space-time processing systems use multiple transmit antennas and time slots to encode information, making use of the variety that the MIMO channel offers to improve communication reliability. Consider a space-time coding system in which the transmitter has $N_{t}$ Antennas, the receiver has $N_{r}$ antennas, and a block of length $N_{s}$ is used [10]. The input-output relationship of the system is governed by the equation.

(5) Y=HC+N

where C represents the transmitted codeword. The structure of C is determined by the specific coding scheme, such as the Alamouti code or other OSTBCs, designed to maximize diversity and minimize error probability in fading channels. The matrix C ∈ ${C\;}^{N_{t}\;x{\;N}_{s}}$ represents the transmitted codeword on each of the $n_{t}$ antennas for each sample, H ∈ ${C\;}^{N_{r}\;x{\;N}_{t}}$ represents the channel coefficients between the transmitter and receiver antennas, and N ∈ ${C\;}^{N_{r}\;x{\;N}_{s}}$ is a matrix of additive noise samples. The structure of the codewords C is determined by the coding scheme used [11].

### 2.3 Real OSTBC

The orthogonality condition is essential for simplifying decoding and ensuring that the transmitted signals can be separated reliably at the receiver. Mathematically, for a transmission matrix X, the orthogonality condition can be expressed as [12]:

(6) $$XX^{T}=\sum_{n=1}^{N_{t}}{\left|S_{n}\right|}^{2}I_{T}$$

where, $I_{T}$ is the identity matrix of size T. This can also be interpreted as the dot product of the columns. $S_{i}\cdot S_{j}=0$ where $S_{i}$, $S_{j}$ are column matrix of X , i≠j. It follows from Ref. [2] that Orthogonal designs are delay optimal with code rate one for n = 2, 4, and 8. It also follows that the construction of complex OSTBC with a code rate of 1 can only be achieved if and only if n = 2 or n = 4. We can't find an orthogonal design for n > 8 with code rate 1 or with full rate, rather a combination matrix of this scheme can help us achieve a rate optimal Real OSTBC design for 16 by 16, 32 by 32, and 64 by 64 antenna configuration or when n = 16, n = 32 and n = 64 with a code rate of 0.5, 0.125 and 0.0625 respectively which can be used for massive MIMO case [13]. Real OSTBC works with real numbers, which leads to simpler operations like real matrix manipulations, which reduces the overall decoding complexity and is also a good extension for larger-size antenna configurations.

$$X_{8}=\left[\begin{array}{cccccccc} z_{1} & z_{2} & z_{3} & z_{4} & z_{5} & z_{6} & z_{7} & z_{8}\\ {-z}_{2} & z_{1} & z_{4} & {-z}_{3} & z_{6} & {-z}_{5} & -z_{8} & z_{7}\\ {-z}_{3} & -z_{4} & z_{1} & z_{2} & z_{7} & z_{8} & -z_{5} & -z_{6}\\ {-z}_{4} & z_{3} & -z_{2} & z_{1} & z_{8} & {-z}_{7} & z_{6} & -z_{5}\\ -z_{5} & {-z}_{6} & -z_{7} & -z_{8} & z_{1} & z_{2} & z_{3} & z_{4}\\ {-z}_{6} & z_{5} & -z_{8} & z_{7} & -z_{2} & z_{1} & -z_{4} & z_{3}\\ -z_{7} & z_{8} & z_{5} & -z_{6} & -z_{3} & z_{4} & z_{1} & -z_{2}\\ {-z}_{8} & {-z}_{7} & z_{6} & z_{5} & -z_{4} & -z_{3} & z_{2} & z_{1} \end{array}\right]$$

$$B_{8}=\left[\begin{array}{cccccccc} {-z}_{1} & {-z}_{2} & -z_{3} & -z_{4} & -z_{5} & -z_{6} & -z_{7} & {-z}_{8}\\ z_{2} & -z_{1} & {-z}_{4} & z_{3} & -z_{6} & z_{5} & z_{8} & -z_{7}\\ z_{3} & -z_{4} & -z_{1} & -z_{2} & -z_{7} & {-z}_{8} & z_{5} & z_{6}\\ z_{4} & z_{3} & z_{2} & -z_{1} & -z_{8} & z_{7} & {-z}_{6} & z_{5}\\ z_{5} & {-z}_{6} & z_{7} & z_{8} & {-z}_{1} & -z_{2} & {-z}_{3} & {-z}_{4}\\ z_{6} & z_{5} & z_{8} & -z_{7} & z_{2} & {-z}_{1} & z_{4} & -z_{3}\\ z_{7} & z_{8} & -z_{5} & z_{6} & z_{3} & -z_{4} & -z_{1} & z_{2}\\ z_{8} & {-z}_{7} & {-z}_{6} & {-z}_{5} & z_{4} & z_{3} & -z_{2} & -z_{1} \end{array}\right]$$

$$X_{16}={\left[\begin{array}{cc} X_{8} & X_{8}\\ B_{8} & X_{8} \end{array}\right]}^{T}$$

$$X_{32}={\left[\begin{array}{cccc} X_{8} & X_{8} & X_{8} & X_{8}\\ B_{8} & X_{8} & B_{8} & X_{8}\\ B_{8} & B_{8} & X_{8} & X_{8}\\ X_{8} & B_{8} & B_{8} & X_{8} \end{array}\right]}^{T}$$

$$X_{64}={\left[\begin{array}{cc} X_{32} & X_{32}\\ B_{32} & X_{32} \end{array}\right]}^{T}$$

### 2.4 Maximum likelihood detection

As discussed above the combiner for OSTBC will have a form of [14].

(7) $$\tilde{x}=Kx+N$$

Here, $\tilde{x}$ is the symbol we want to detect, x is the channel gain values of the transition probability matrix, N is a noise term, which we consider to be complex Gaussian with zero mean and variance, and is the output of the combiner. Additionally, suppose that the M-ary alphabet A is the source of the symbol being sent. The maximum a-posteriori probability estimates of x, which is the maximum likelihood criterion denoted by s can be expressed as [14]

(8) $$s=\underset{\left\{x\right\}}{\arg\;\max}P\left(\tilde{x}|x\right)$$

Decision Rule

(9) $$s=\underset{\left\{x\right\}}{\arg\;\min}\left[{\left|\tilde{x_{i}}-x_{i}\right|}^{2}+\sum_{t=1}^{N_{T}}\sum_{j=1}^{N_{R}}{\left|h_{j,t}\right|}^{2}-1)\;{\left|x_{i}\right|}^{2}\right]$$

### 2.5 Pairwise error probability

The PEP is the probability of erroneously decoding the codeword $C^{1}$ and $C^{2}$, denoted by $P\left(C^{1}\to \;C^{2}\right)$. Suppose the codebook contains M code words then the upper bound on the probability of error that code word $C^{1}$ was transmitted and erroneously decoded [14].

(10) $$P\left(error\to \;C^{1}\right)\leq \sum_{i=2}^{N_{R}}P\left(C^{1}\to \;C^{i}\right)$$

Assuming a known channel matrix *H*, we now compute the PEP for a Rayleigh fading channel. To compute the average error, the distribution of the errors will be averaged across H [15].

(11) $$P\left(error\to \;C^{1}\right)\leq \frac{1}{2}\;{\left[\frac{1}{\prod_{j=1}^{N_{t}}\left(1+\frac{\rho }{4}\;\lambda_{j}\right)}\right]}^{N_{r}}$$

where $\lambda_{j}$ are the eigenvalues of the matrix H, ρ is the average energy per symbol, please refer to Appendix Section II for more explanation. Since diversity gain is defined as the slope of the decoding error versus signal-to-noise ratio curve at large signal-to-noise ratios, we consider the PEP bounds in the limit as ρ→∞.In this limit, the Rayleigh PEP bound is given by the following [15]

(12) $$\lim_{\rho \to \infty }P\left(error\to \;C^{1}\right)\leq \frac{1}{2}\;{\left[\frac{1}{4}{\left(\prod_{j=1}^{r}\lambda_{j}\;\right)}^{1/r}\rho \;\right]}^{{-rN}_{r}}$$

As L increases to 2, 4, or 8 … blocks, which increases the $N_{t}$, the receiver starts to accumulate more diversity and redundancy from the transmitted blocks. Each additional block allows the receiver to use more parity symbols or new combinations of the original data, which improves the PEP.

## 3 Rateless OSTBC for massive mimo communication system

### 3.1 Concept of fountain codes

Fountain codes are a great option for dependable transmission in wireless communication. They are often referred to as rateless or rate-adaptive codes. The term “fountain” is derived from the analogy of a water fountain where droplets are continuously produced, and the destination bucket fills up as long as it receives enough droplets, regardless of the exact sequence. Similarly, in wireless communications, fountain codes generate and transmit a potentially unlimited number of encoded packets until the receiver accumulates enough mutual information to decode the original message.

Consider a source that generates blocks of data packets, which need to be transmitted reliably. This source block is divided into equal partitions, known as source symbols [16]. To ensure effective forward error correction, these K source symbols are encoded, allowing the system to maintain reliability even if some packets are lost during transmission. In a wireless communication environment, factors such as interference, noise, path loss, and atmospheric conditions can degrade signal quality and transmission accuracy. Fountain codes address these challenges by continuously generating encoded symbols until the receiver, equipped with an optimal rateless decoder, acknowledges that it has received enough packets to successfully recover the original data.

On the vector space ${Ϝ}_{2}^{k}$. Fountain codes are determined by a probability distribution D. As per reference [23] the encoding process that yields an encoded symbol $x_{j}$ is as follows:

1. **Sampling**: Sample the distribution D to obtain a vector ($a_{1},a_{2},\ldots ,a_{k})$ ∈ ${Ϝ}_{2}^{k}$.
2. **Calculation**: Calculate the encoded symbol $x_{j}$ using the formula $x_{j}=\sum_{i=1}^{k}a_{i}s_{i}$.

### 3.2 Rateless Orthogonal Space Time Block Codes

A critical component in enhancing the fruit of MIMO systems is the use of codes, which can tackle the adverse effects of noise, CSI, and path loss in wireless channels. Among these, rateless codes and space-time codes (STCs) have gained significant attention. A sort of FEC code called a rateless code, also called a rate adaptable code, can produce an infinite amount of encoded symbols from a given set of input symbols. Rateless codes do not need a pre-established code rate, in contrast to conventional fixed-rate codes. Instead, the transmitter continues to send encoded symbols until the receiver acknowledges successful decoding. This adaptability makes rateless codes particularly effective in varying channel conditions, as they can dynamically adjust to the channel quality without the need for retransmissions.

Combining rateless codes with STCs can provide an additional layer of error correction, further improving the reliability of MIMO communication systems. While STCs exploit spatial and temporal diversity, rateless codes can adapt to varying channel conditions, ensuring that the receiver can decode the message even in challenging environments. Additionally, this improved spectral efficiency and robustness to changing channel conditions in mobile users can be leveraged by combining these technologies.

To achieve Rateless behavior with OSTBC, we adapt the transmission scheme such that the number of transmitted symbols can vary based on the channel conditions. This can be described mathematically by considering the mutual information between the transmitter and the receiver and comparing it with the required threshold for successful decoding.

The mutual information I between the transmitter and receiver can be expressed as [17]:

(13) $$Y_{l}=\sqrt{\frac{P}{N_{T}\;}}\;H_{l}X_{l}+N_{l}$$

(14) $$I\left(X_{l};Y_{l}|H_{l}\right)=H\left(Y_{l}|H_{l}\right)-H\left(Y_{l}|X_{l},H_{l}\right)$$

Equation (14) is the definition of mutual information. It represents the amount of information that the received signal. $Y_{l}$ contains the transmitted signal $X_{l}$ given the channel state $H_{l}$.

(15) $$H\left(y\right)=\log_{2}\;\det\left(\pi eK\right)$$

Equation (15) represents the entropy of a multivariate Gaussian random variable y with covariance matrix K. The entropy H(y) is a measure of uncertainty or information content. The determinant of K determines the spread of the distribution.

(16) $$K_{Y_{l}}=H_{l}H_{l}^{H}\frac{P}{N_{t}}+I_{N_{r}}$$

The above equation (16) represents the covariance matrix of the received signal $Y_{l}$. Here, $H_{l}H_{l}^{H}$ is the covariance matrix of the channel $H_{l}$, $\frac{P}{N_{t}}$ is the transmit power normalized by the number of transmit antennas? $N_{t}$ , and $I_{N_{r}}$ is the identity matrix corresponding to the number of receive antennas $N_{r}$.

(17) $$K_{Y_{l}|X_{l}}=I_{N_{r}}$$

This represents the covariance matrix of the received signal $Y_{l}$ conditioned on the transmitted signal $X_{l}$. Under the assumption of additive white Gaussian noise (AWGN), this covariance matrix is just the identity matrix $I_{N_{r}}$ , indicating that the noise is uncorrelated and has unit variance

(18) $$H\left(Y_{l}|H_{l}\right)=\log_{2}\;\det\left(\pi eK_{Y_{l}}\right)$$

The above equation (18) computes the entropy of the received signal $Y_{l}$ given the channel $H_{l}$. The term $K_{Y_{l}}$ is the covariance matrix from equation (17). Substituting equation (17), (18)

(19) $$H\left(Y_{l}|X_{l},H_{l}\right)=\log_{2}\;\det\left(\pi eI_{N_{r}}\right)$$

This equation computes the entropy of the received signal $Y_{l}$ given both the transmitted signal $X_{l}$ and the channel $H_{l}$. Since the covariance matrix $K_{Y_{l}|X_{l}}$ is the identity matrix $I_{N_{r}}$, the entropy simplifies to $N_{r}\;\log_{2}\left(\pi e\right)$ [18].

Substituting the results from equations (18), (19) into the mutual information definition (14), we get this final expression. It shows that the mutual information is determined by the eigenvalues of the matrix $H_{l}H_{l}^{H}$ scaled by the signal-to-noise ratio $\frac{P}{N_{t}}$ , where P is the signal-to-noise ratio (SNR), $H_{l}$ is the channel matrix for the l-th block, $N_{t}$ is the number of transmit antennas, and L is the number of blocks.

(20) $$I\left(X_{l};Y_{l}\right)=\sum_{l=1}^{L}\;\log_{2}\;\det\;\left(I_{N_{T}}+\frac{P}{N_{T}\;}H_{l}H_{l}^{H}\right)$$

The receiver attempts to decode the message after receiving each block, equation (20) is explained in the appendix section. If the accumulated mutual information I is greater than or equal to the required threshold for decoding the message RLT (where R is the rate, L is the number of blocks, and T is the number of time slots per block), the receiver decodes the message. Mathematically, this condition is [17]:

(21) I≥RLT

If this condition is met after receiving the l-th block, the receiver decodes the message and sends feedback to the transmitter to stop further transmission. If the mutual information I is less than RLT after the l-th block, the receiver waits for the next block. This process continues until the mutual information is sufficient for decoding.

The received signal matrix Y for the l ^th^ block can be represented as [19]:

(22) $$Y_{l}=H_{l}X_{l}+N_{l}$$

where $H_{l}$ is the channel transition matrix for the l-th block transmission and $N_{l}$ is the noise matrix for the l-th block transmission.

The mutual information for each block is calculated and accumulated [20]:

(23) $$I\left(X_{i};Y_{i}\right)=\sum_{l=1}^{L}\;\log_{2}\;\det\;\left(I_{N_{T}}+\frac{P}{N_{T}\;}H_{l}^{H}H_{l}\right)$$

(24) $$I_{l}=\log_{2}\;\det\;\left(I_{2}+\frac{P}{N_{T}\;}H_{l}^{H}H_{l}\right)$$

The receiver decodes the message if I≥RLT. The receiver sends a positive feedback signal to the transmitter, indicating that decoding was successful, and the transmitter stops further transmission of the current message.

## 4 Simulation and results

**Fig. 1:**
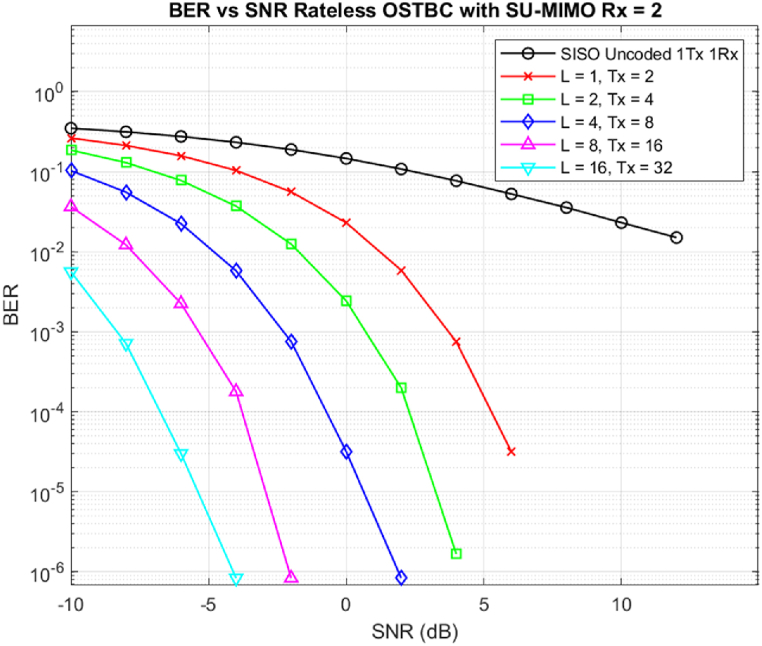
Rateless OSTBC over different transmitted block Lengths.

The following simulation was done for ROSTBC as shown in Fig. 1 to showcase how these codes achieve channel condition adaptivity and their robust nature across low SNR values. In order to simulate ROSTBC, a Real Orthogonal 2 × 2 antenna group was used in which the transmit antenna number will increase as a multiple of L blocks used depending on the channel condition.

During simulation open loop MIMO communication was used in which case, channel state information is known at the receiver but not at the transmitter. During modulation BPSK modulation across real OSTBC was used to encode each block of information. The signal was able to propagate through the Rayleigh fading channel with AWGN noise added during transmission. As shown from the figure when L = 1 ROSTBC will not perform well since the decoder will not have enough information to decode the message across the range of SNR values.

When L increases from L = 2 up to L = 16 we can notice that the performance will greatly improve. This happens because whenever the channel condition is harsh or we are not able to decode the transmitter will increase the number of L blocks that will be used. This group of blocks will inherit $LN_{t}$ Transmit antennas compensate effect of delay and diversity gained from using multiple antennas. Values of L are not fixed for these scenarios since this depends on the channel condition which makes the channel codes become Rateless and rate adaptive to channel conditions.

**Fig. 2:**
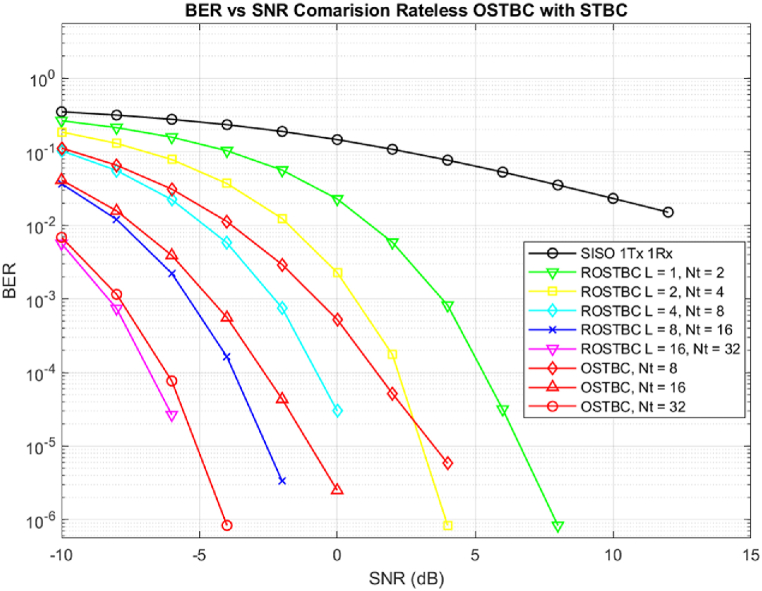
Comparison of ROSTBC with static OSTBC for SU-Massive MIMO with 2 receive antennas.

Comparison in Performance of ROSTBC and static rate OSTBC is shown Fig. 2 shows simulation for ROSTBC over the range of SNR values from −10db to 10 db over Rayleigh fading channel. As expected ROSTBC will have low performance for L = 1 and L = 2 to achieve a comparison state to static 8 by 8 OSTBC. When L is increasing the performance will match and also surpass the performance of 16 by 16 and 32 by 32 OSTBC Massive MIMO Communication scheme.

Table 1 illustrates numerical data found from the simulation of Fig. 2, showing that ROSTBC is robust in matching the channel condition and achieving a good BER with very low SNR values. ROSTBC with L = 16 will inherit 16 × 2 antenna groups to achieve 32 transmit antennas outperforming the static OSTBC with a BER value of $7.928x10^{-5}$, this was achieved when the channel state information was not known at the transmitter.

**Table 1:** Numerical analysis of BER vs SNR for ROSTBC over a different number of encoding blocks in comparison to OSTBC with 2 receive antennas.

Antenna Configuration	Number of Blocks	BER at −10dB	BER at -8dB	BER at -5dB	BER at 0 dB	BER at 2 dB	BER at 5 dB
$N_{t}=2$	L=1	0.2642	0.2132	0.1579	0.1131	0.00593	$8.834x10^{-5}$
$N_{t}=4$	L=2	0.1855	0.05543	0.03721	0.00228	$1.58x10^{-4}$	–
$N_{t}=8$	L=4	0.10295	0.05543	0.02251	$7.1x10^{-4}$	–	–
$N_{t}=16$	L=8	0.03671	0.0121	0.0023	$1.7x10^{-4}$	–	–
$N_{t}=32$	L=16	0.00572	0.00069	$7.928x10^{-5}$	–	–	–
OSTBC $N_{t}=8$	–	0.1123	0.0659	0.0114	0.0005	$1.794x10^{-4}$	$8.155x10^{-6}$
OSTBC $N_{t}=16$	–	0.0415	0.0156	0.0039	$1.1144\;x10^{-4}$	$8.1548\;x10^{-6}$	–
OSTBC $N_{t}=32$	–	0.0069	0.00112	0.0011	$1.0927x10^{-4}$	$5.4366\;x10^{-6}$	–

Comparing equalizing the number of transmit antennas used in Rateless OSTBC to match with that of static OSTBC for 8, 16, and 32 transmit antennas in the case of Massive MIMO we can say that we can improve BER with 8.3 % in 8 transmit, 11.5 % in 16 transmit antenna scheme and 39.1 % in 32 transmit antennas.

**Fig. 3:**
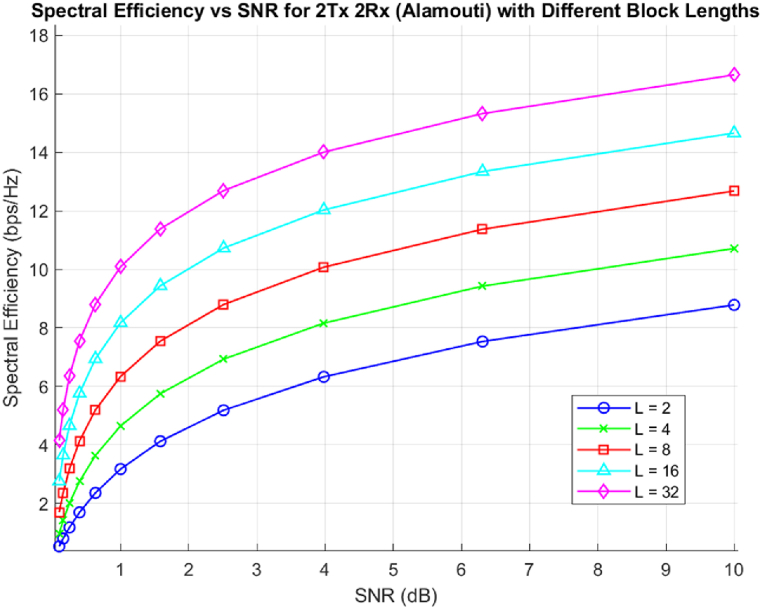
Spectral Efficiency of Rateless OSTBC over different number of blocks used.

Fig. 3 shows that the spectral efficiency of Rateless OSTBC will improve as the number of blocks utilized increases since the number of transmit antennas with the robustness of the code also improves during signal encoding and propagation. Even though this encoding scheme shows a good spectral efficiency it might not be as that of static OSTBC due to the nature of L blocks utilized will affect the diversity multiplexing tradeoff and also the code rate of encoding blocks.

**Fig. 4:**
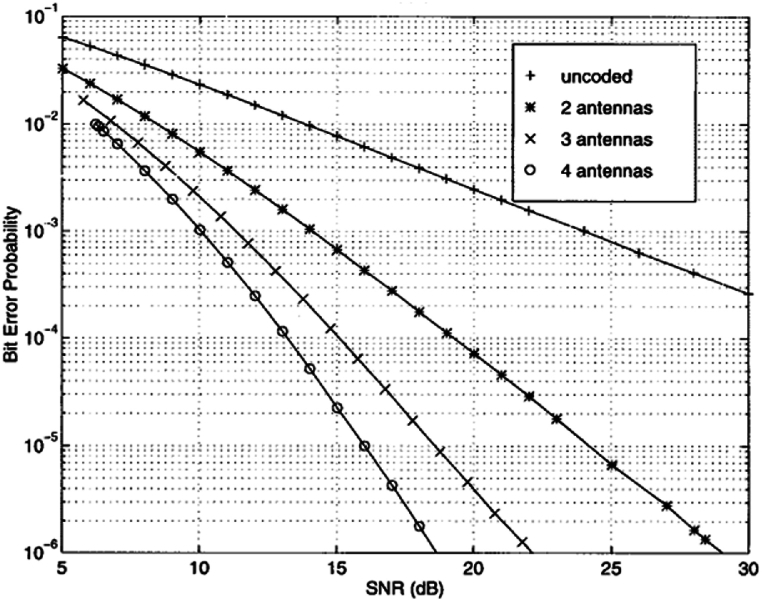
Bit Error Probability vs SNR STBC with 1 receive antenna from Tarokah's Performance analysis work for G4 [2].

**Fig. 5:**
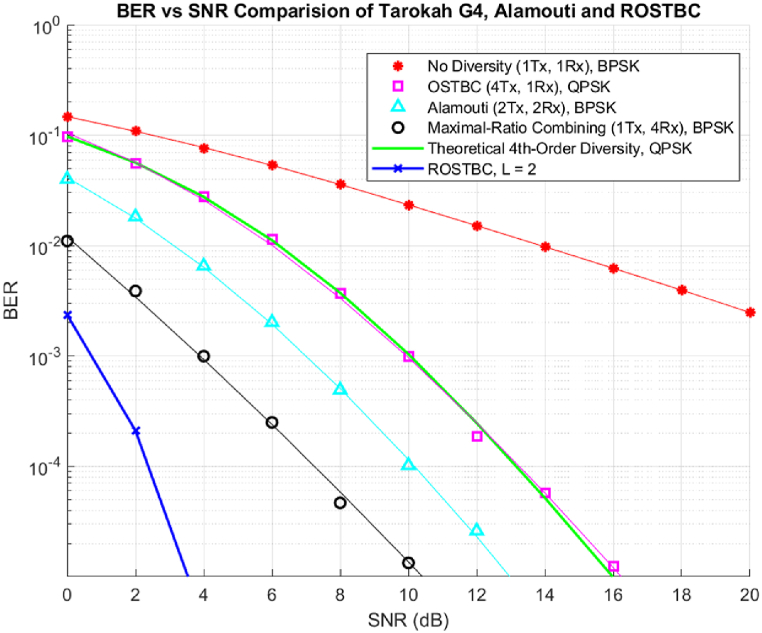
Simulation result of OSTBC as compared to previous research finding static STBC.

Fig. 4 shows the previously experimented simulations for static space-time block codes for G4 space-time encoding. These research works were baselines for space-time block codes, next we will compare ROSTBC with these findings by integrating the encoding (see Fig. 5).

As expected, the simulation result shown in Fig. 4.11 outputs that a Rateless OSTBC at L = 2, will match the rate adaptability condition and continue to operate within that state. The above shows that compared to previous works of static STBC this rate adaptive codes perform well with low bound of SNR and good BER creating reliability in the scheme of MIMO communication.

## 5 Conclusion

In conclusion, this work has successfully developed and implemented robust Rateless Space-Time Block Codes (ROSTBC) tailored for mmWave Massive MIMO communication systems, addressing the key challenge of reliable communication under uncertain channel conditions. By revisiting and building on the core concepts of MIMO and massive MIMO communication, we have demonstrated the significant potential of large antenna arrays to enhance spectral efficiency and link reliability. The integration of rateless coding with OSTBC allows the system to dynamically adapt to unknown channel state information (CSI), providing a flexible and resilient solution in the face of varying channel conditions.

Through both theoretical analysis and extensive simulations, we compared the performance of the proposed ROSTBC with traditional STBCs, revealing considerable improvements in error performance and diversity gain, especially in low SNR scenarios. These findings highlight the advantages of rateless coding for mmWave communications and underscore its potential to improve reliability and efficiency in next-generation wireless networks.

In bit error rate (BER) performance, particularly in challenging channel environments such as the Rayleigh fading channel and addressing the concerns of mmWave Massive MIMO.

## CRediT authorship contribution statement

**Zelalem A. Kelem:** Writing – original draft, Visualization, Software, Methodology, Formal analysis, Conceptualization. **Habib M. Hussein:** Writing – review & editing, Visualization, Validation, Supervision, Resources, Methodology, Data curation, Conceptualization.

## Declaration of competing interest

I am submitting a manuscript for consideration for publication in Heliyon journal. The manuscript is entitled **“*Robust Rateless Space Time Block Coding for mmWave Massive MIMO System*”.** It has not been published elsewhere and it has not been submitted simultaneously for publication elsewhere. However, it has been submitted to Heliyon journal with Article ID: HELIYON-D-24-55126 and got a comment for revising the article. Now, the article is revised by attempting all the comments of the reviewers on a line-by-line basis.

In the rapidly evolving landscape of wireless communication, ensuring reliability and robustness in encoding mechanisms for mmWave Massive MIMO systems is crucial. Our research investigates space-time block codes (STBC) as a solution to manage uncertainty and enhance reliability in such systems. This paper offers an in-depth review of key concepts including MIMO communication, Massive MIMO communication, space-time block codes, and rateless coding within the framework of wireless systems.

Building upon information theory and mmWave Massive MIMO principles, we developed space-time block codes that preserve orthogonality for real-valued symbols. After thoroughly analyzing these codes, we extended them to Rateless Orthogonal Space-Time Block Codes (ROSTBC), which adapt their rates dynamically to varying channel conditions. Our research concludes with a comparative analysis of G4-encoded Tarokh work and other Orthogonal Space-Time Block Codes (OSTBCs) versus ROSTBCs. The results demonstrate that ROSTBCs outperform static OSTBCs by at least 8.5 % in very low SNR scenarios as the number of blocks increases.

This research highlights the potential of ROSTBCs to improve reliability in wireless communication systems by providing adaptive solutions to traditionally unpredictable environments.

## Appendix

### Section I

$$H\left(Y_{l}|H_{l}\right)=\log_{2}\;\det\left(\pi e\left(H_{l}H^{H_{l}}\frac{P}{N_{t}}+I_{N_{r}}\right)\right)$$

$$H\left(Y_{l}|X_{l},H_{l}\right)=\log_{2}\;\det\left(\pi eI_{N_{r}}\right)=N_{r}\;\log_{2}\left(\pi e\right)$$

$$I\left(X_{l};Y_{l}|H_{l}\right)=\log_{2}\;\det\left(\pi e\left(H_{l}H_{l}^{H}\frac{P}{N_{t}}+I_{N_{r}}\right)\right)-N_{r}\;\log_{2}\left(\pi e\right)$$

$$I\left(X_{l};Y_{l}|H_{l}\right)=\log_{2}\;\det\left(H_{l}H_{l}^{H}\frac{P}{N_{t}}+I_{N_{r}}\right)-N_{r}\;\log_{2}\left(\pi e\right)+N_{r}\;\log_{2}\left(\pi e\right)$$

$$I\left(X_{l};Y_{l}\right)=\sum_{l=1}^{L}\;\log_{2}\;\det\;\left(I_{N_{T}}+\frac{P}{N_{T}\;}H_{l}H_{l}^{H}\right)$$

### Section II

In Rayleigh fading, the elements of H are independent, zero-mean, complex Gaussian random variables with variance equal to 1/2 per dimension. Thus,

$$\beta_{i,j}=\sum_{k=1}^{N_{t}}u_{k,j}h_{i,k}∼CN\left(0,1\right)\text{,}$$

Then the distribution of ${\left|\beta_{i,j}\right|}^{2}$ can be given as

$$f_{{\left|\beta_{i,j}\right|}^{2}}\left(y\right)=\frac{1}{2\sigma^{2}}e^{-y/{2\sigma }^{2}}$$

Following the above the distribution of ${\left|\beta_{i,j}\right|}^{2}$ the unconditional PEP upper bound is given by

$$P\left(error\to \;C^{1}\right)\leq \frac{1}{2}\int_{0}^{\infty }P\left(error\to \;C^{1}\right)\;f_{{\left|\beta_{i,j}\right|}^{2}}\;\left(y\right)dy$$

$$P\left(error\to \;C^{1}\right)\leq \frac{1}{2}\int_{0}^{\infty }\prod_{i=1}^{N_{r}}\prod_{j=1}^{N_{t}}\exp\left(-\frac{\rho }{4}\;\lambda_{j}{\left|\beta_{i,j}\right|}^{2}\right)\exp\left(-{\left|\beta_{i,j}\right|}^{2}\right)d{\left|\beta_{i,j}\right|}^{2}$$

$$P\left(error\to \;C^{1}\right)\leq \frac{1}{2}\int_{0}^{\infty }\prod_{i=1}^{N_{r}}\prod_{j=1}^{N_{t}}\exp\left(-\frac{\rho }{4}\;\lambda_{j}y\right)\exp\left(-{\left|\beta_{i,j}\right|}^{2}\right)e^{-y}\;dy$$

$$P\left(error\to \;C^{1}\right)\leq \frac{1}{2}\prod_{i=1}^{N_{r}}\prod_{j=1}^{N_{t}}\left(\frac{1}{1+\frac{\rho }{4}\;\lambda_{j}}\right)$$

$$P\left(error\to \;C^{1}\right)\leq \frac{1}{2}\;{\left[\frac{1}{\prod_{j=1}^{N_{t}}\left(1+\frac{\rho }{4}\;\lambda_{j}\right)}\right]}^{N_{r}}$$

Where $\lambda_{j}$ are the eigenvalues of the matrix H, ρ is the average energy per symbol.
